# Can a brief psychological intervention improve oral health behaviour? A randomised controlled trial

**DOI:** 10.1186/s12903-018-0627-y

**Published:** 2018-10-03

**Authors:** U. Wide, J. Hagman, H. Werner, M. Hakeberg

**Affiliations:** Department of Behavioral and Community Dentistry, Institute of Odontology, The Sahlgrenska Academy, University of Gothenburg, P.O. Box 450, SE-40530 Gothenburg, Sweden

**Keywords:** Acceptance and commitment therapy, Cognitive behaviour therapy, Psychological intervention, Caries, Young adults, Oral health behaviours, Randomised controlled trial

## Abstract

**Background:**

Dental caries is a major public health issue affecting a large proportion of the general population**.** The disease is associated with behavioural factors and is thus preventable to a high degree. Individuals may need assistance to be able to change their oral health behaviour. There is a lack of such interventions for adults affected by severe caries. The aim of the study was to evaluate the effect of Acceptance and Commitment Therapy (ACT), a form of cognitive behavioural therapy, on oral health behaviour in young adults with poor oral health.

**Methods:**

The study included a two group parallel randomised controlled trial at general dental clinics, with young adults, 18–25 years of age, ≥ two manifest proximal dental caries lesions (*n* = 135); 67 were treated with ACT and 68 with standard disease information only, respectively. Primary outcomes: oral health behaviours (tooth-brushing, flossing, use of toothpicks, and additional fluoride use). The CONSORT principles for RCTs were used, including intention-to-treat and per protocol analyses. The Chi-square, Mann-Whitney, and Wilcoxon Signed Rank tests were applied, including effect sizes.

**Results:**

The study groups did not differ with regard to oral health behaviour variables at baseline. The intervention group improved all their oral health behaviours significantly over time (effect sizes, 0.26–0.32), while the control group showed improved behaviours on two measures (flossing and additional use of fluoride, effect sizes, 0.22–0.23).

**Conclusions:**

By testing a psychological intervention on young adults (18–25 years of age) with a high prevalence of caries, we found an immediate positive effect with improved oral health behaviours.

**Trial registration:**

TRN ISRCTN15009620, retrospectively registered 14/03/2018.

## Background

Dental caries is a major public health issue, affecting around 60–90% of children, adolescents and adults worldwide [1]. Dental caries is associated with negative consequences and costs to sufferers and oral care providers [2, 3]. Moreover, dental caries is largely related to behavioural factors, such as oral hygiene, fluoride exposure and dietary habits. Thus, dental caries may be treated and prevented with behavioural interventions at the individual level.

Recent research in public health stresses the social determinants of oral health and inequalities in health, and the need for structural interventions to improve health and reduce health inequalities [4–6]. However, the dental care practice also needs effective methods to help individuals with poor oral health to change their behaviour.

One recent systematic review found evaluations and, to some degree, positive effects of behavioural interventions for adult individuals in the field of dentistry, mainly for older adults affected by periodontitis (besides caries, the other major oral disease) [7]. Similar findings were reported by Newton and Asimakopoulou for behaviour interventions in improving oral hygiene related behaviour in patients with periodontitis [8]. However, a systematic review found no behavioural interventions for dietary change in adult patients with dental caries [9]. Thus, less is known about behavioural interventions for adults affected by caries. Young adults develop health behaviour habits for their adult lives, and possibly for their children, and are therefore an important group to target for behavioural interventions. Authors have emphasised the need to use stringent interventions based on accepted theory from the behavioural sciences (the field of health psychology) to affect oral health behaviour changes [10, 11].

Different theory-based interventions for behaviour change has been developed, and to some degree tested for oral health problems. The present study was designed to evaluate a brief psychological intervention, Acceptance and commitment therapy (ACT), delivered by a psychologist in general dentistry, as a means to help young adult patients with poor oral health to make behavioural changes to improve their oral health.

ACT is a recently developed psychological method [12], a form of Cognitive Behaviour Therapy (CBT), that has been used with positive results in the treatment of health problems, such as pain, tinnitus and addiction [13, 14]. ACT interventions have been developed in brief formats for primary care, a setting similar to general dentistry, but has, to our knowledge, not been tested in general dentistry. The rationale of ACT is to increase psychological flexibility, thus facilitating the individual to maintain functional behaviours, and to change dysfunctional behaviours, in order to live in accordance with chosen individual life values [15]. The intention of using ACT in the present study was to contribute to behaviour change by focusing on how health-related behaviour could be relevant to valued life directions, by addressing also the psychological flexibility of the individual.

## Methods

### The aim and design of the study

The aim of the study was to evaluate the effect of ACT on oral health behaviours in young adults with poor oral health. Hypothesis: A brief psychological intervention (ACT) improves oral health behaviours (such as tooth-brushing and flossing) more than standard information alone.

The present analysis is part of a larger clinical trial evaluating the effect of ACT on oral health behaviours, oral health (caries, gingivitis), sugar consumption, psychological distress, general health behaviour and the ability to handle stress. In the present analysis the primary outcomes were oral health behaviours (flossing, toothpick use, tooth-brushing, additional fluoride use).

The study was a two group parallel randomised controlled trial with an allocation ratio 1:1. The study was approved by the Regional Ethical Review Board in Gothenburg (Reg. no. 840–12).

### Participants

The participants were recruited between 2013 and 2014 at two Public Dental Service clinics in Region Västra Götaland, Sweden. Inclusion criteria: 18–25 years of age, ≥ two manifest proximal dental caries lesions. Exclusion criteria: Psychiatric/neuropsychiatric diagnosis, such as depression, psychosis, autism spectrum disorder, mental retardation, substance abuse. Participants needed to have good understanding of Swedish which was assessed by the research coordinator. A power analysis was performed to determine the sample size. The calculation was made for gingivitis (mean ratio of bleeding surfaces) and the assumption of detecting a 20% reduction with an alpha of 0.05 and a power of 0.80. The number of participants needed was 53 individuals per group. Thus, including dropouts, the sample was determined to require at least 130 participants, 65 per group. Power calculations were repeated with other outcome variables (plaque, caries, oral health behaviours), but these did not change the minimum number of participants needed to detect a relevant difference between groups.

**Fig. 1:**
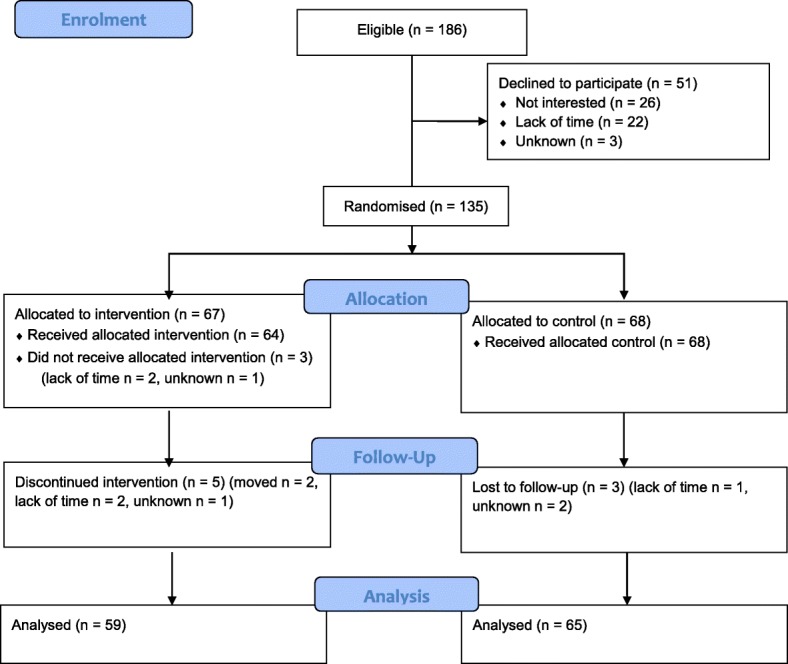
Flow diagram of the progress through the phases of the Intervention group and Control group: Enrolment, intervention allocation, follow-up and data analyses

Potentially eligible individuals were screened (first screening) while at their ordinary routine dental examination, and were invited to participate in the trial. The research coordinator at the dental clinic contacted individuals interested in participating, and after a second screening/confirmation of the inclusion/exclusion criteria, the individual received written information about the trial. The study participants were asked for and provided written consent. The second screening resulted in 186 eligible patients. Of these, 51 declined to participate, the most common reasons being “not interested” and “lack of time” (see Fig. 1). The final sample consisted of 135 participants (acceptance rate 72.6%).

### Procedure and allocation strategy

Individuals included in the study answered baseline questionnaire using a touch-screen computer. Clinical data were obtained from their most recent ordinary dental examination. All participants then received standardised oral health information, provided verbally by a registered dental nurse using a brochure on oral health behaviour and caries. The information, including the brochure, was at the time of the study used at all public dental service clinics in Region Västra Götaland, Sweden.

The participants were then randomised by an independent research coordinator, either to the Intervention (ACT plus information) or Control (information alone) group, using a block randomisation procedure including stratification by gender and smoking (randomly permuted blocks within strata [16]). As an allocation strategy, the research coordinator used sealed opaque envelopes that had been prepared in advance by another research coordinator and placed in four boxes according to the stratification strategy. The allocation list was kept in a locked safety box, only available to the independent research coordinator.

Participants allocated to intervention were scheduled for two appointments with the psychologist 2 weeks apart (see below for description of the intervention). Participants answered follow-up questions at the clinic 3 weeks after baseline. (See Fig. 1, Flow chart according to CONSORT [17]).

At the two dental clinics, the study involved general practitioners (dentists and dental hygienists), a research coordinator, dental nurses and a clinical psychologist. All treatments and examinations were performed at the respective clinic.

### Measures

Clinical measure of oral health: data on dental caries lesions (number and type) according to accepted standards (D1-D3, secondary caries), including assessment of caries on four surfaces, with proximal caries assessed on radiographs [18]. A summarised score of the number of surfaces with manifest caries (D3 and secondary caries) was calculated.

Sociodemographic characteristics were measured with questions about: age, gender, ethnicity (Swedish-born, including other Nordic country; foreign-born), mother’s country of birth (Swedish-born, including other Nordic country; foreign-born), housing (rented flat; own flat/house; other), mother’s education (primary; secondary; university).

Self-rated oral health was captured with the question ‘How do you rate your oral health?’, with four response alternatives (poor; fair; good; very good).

Oral health behaviour was assessed with questions about tooth-brushing, flossing, use of toothpicks, and use of additional fluoride (besides toothpaste), with six response alternatives: three times a day or more; twice a day; once a day; several times a week; once a week; more seldom/never. One question measured dental care attendance, where the five response alternatives were dichotomised into often (twice a year; once a year) vs. seldom (every other year; less then every other year; only when acute problem).

Any adverse effects during the study period reported by the participants were registered by the research coordinators.

### Intervention

The intervention used was a psychological intervention, CBT in the ACT form [12, 15], adapted to primary care settings [19, 20] and modified for the present trial. The modification included a selection of well-known ACT exercises (e.g., defusion and Bull’s Eye), and was made in close collaboration with a licensed psychologist specialised in ACT and experienced in implementing ACT in primary care. Like other CBT-interventions, ACT is based on an individual case conceptualisation and a functional analysis of behaviour, and the participant and the psychologist together develop a plan for behaviour change. In Table 1 a treatment overview is provided, showing the different ACT modules.

**Table 1:** Treatment overview of ACT for patients with dental caries

Session 1	Session 2
Introduction	Follow-up
Brief interview	Bull’s-Eye
Mindful oral health	Mindful oral health
Focused questions	Value based living
Case conceptualisation	Defusion exercises
Bull’s-Eye	Plan for behavioural change and follow-up
Clarification of values	
Plan for behavioural change	

The intervention was delivered at two general dental clinics and included two individual sessions (45 min each) with a licensed psychologist specialised in ACT (HW). To secure adherence to treatment, the psychologist in the project was regularly supervised [21]. The time between the first and the second session was 2 weeks.

### Statistical analyses

Descriptive statistics used were frequencies, mean, median and standard deviation (SD). The statistical methods applied were the Chi-square test, the Mann-Whitney test for independent groups, and the Wilcoxon Signed Rank test for dependent groups. Both intention-to-treat (ITT) and per protocol (PP) analyses were performed according to the CONSORT principles [17]. The effect size according to Cohen’s ES was calculated for changes over time, applying the Wilcoxon Signed Rank test using the formula z/√*N*, where z is the test statistic and N equals twice the number of individuals included in the respective analyses [22]. According to Cohen’s criteria [23], an effect size around 0.1 = low effect, 0.3 = medium effect, and 0.5 = large effect. The significance level applied was 0.05. Bonferroni corrections for multiple comparisons were applied giving *p*-values for statistical significance of *p* < 0.005 for baseline (Table 2), and *p* < 0.003 for primary outcomes (Table 3).. The study included a blinded design with the research group and statistical analyst being blinded to which treatment was allocated to which patient.

**Table 2:** Sociodemographic and clinical characteristics of participants (*n* = 135) allocated to Intervention or Control, at baseline

Variable	Intervention (*n* = 67)	Control (*n* = 68)	P
Age in years, Mean (SD)	20.4 (2.1)	20.8 (2.2)	ns.
Self-rated oral health, n (%)			ns.
Poor	27 (40.3)	25 (36.8)	
Fair	31 (46.3)	31 (45.6)	
Good	9 (13.4)	12 (17.6)	
Very good	0	0	
Caries, Mean (SD) Median	6.3 (5.2) 4	4.9 (3.7) 5	ns.
Dental care attendance, n (%) often	58 (86.6)	56 (82.4)	ns.
Gender, n (%) female	32 (47.8)	32 (47.1)	ns.
Smoker, n (%) smoking	23 (34.3)	24 (35.3)	ns.
Ethnicity, n (%) Swedish-born	55 (82.1)	48 (70.6)	ns.
Housing, n (%)			ns.
Rental flat	32 (47.8)	33 (48.5)	
Own flat/house	28 (41.8)	25 (36.8)	
Other	7 (10.4)	10 (14.7)	
Mother’s ethnicity, n (%) Swedish-born	44 (65.7)	29 (42.6)	*p* < 0.01^a^
Mother’s education, n (%)			ns.
Primary	15 (22.4)	22 (32.4)	
Secondary	35 (52.2)	31 (45.6)	
University	17 (25.4)	15 (22.1)	

Chi-square (Mann-Whitney for caries), ^a^ Ns after Bonferroni correction

**Table 3:** Oral health behaviour of the participants allocated to Intervention or Control, at baseline and follow-up, according to Per Protocol (PP) and Intention-To-Treat (ITT) analyses, respectively

Variable	Baseline				Follow-up			
	Intervention		Control		Intervention		Control	
	ITT (*n* = 67)	PP (*n* = 59)	ITT (*n* = 68)	PP (*n* = 65)	ITT (*n* = 67)	PP (*n* = 59)	ITT (*n* = 68)	PP (*n* = 65)
Tooth-brushing								
≥ 3 times/day	1 (1.5)	1 (1.7)	2 (2.9)	2 (3.1)	2 (3.0)	2 (3.4)	3 (4.4)	3 (4.6)
Twice a day	37 (55.2)	32 (54.2)	43 (63.2)	42 (64.6)	50 (74.6)	45 (76.3)	46 (67.6)	45 (69.2)
Once a day	16 (23.9)	14 (23.7)	13 (19.1)	12 (18.5)	8 (11.9)	6 (10.2)	9 (13.2)	8 (12.3)
Several times/week	8 (11.9)	7 (11.9)	9 (13.2)	8 (12.3)	6 (9.0)	5 (8.5)	9 (13.2)	8 (12.3)
Once a week	4 (6.0)	4 (6.8)	1 (1.5)	1 (1.5)	1 (1.5)	1 (1.7)	1 (1.5)	1 (1.5)
More seldom/never	1 (1.5)	1 (1.7)	0	0	0	0	0	0
Flossing								
≥ 3 times/day	0	0	0	0	2 (3.0)	2 (3.4)	2 (2.9)	2 (3.1)
Twice a day	7 (10.4)	6 (10.2)	5 (7.4)	5 (7.7)	13 (19.4)	12 (20.3)	7 (10.3)	7 (10.8)
Once a day	4 (6.0)	4 (6.8)	10 (14.7)	10 (15.4)	11 (16.4)	11 (18.6)	14 (20.6)	14 (21.5)
Several times/week	14 (20.9)	11 (18.6)	11 (16.2)	10 (15.4)	13 (19.4)	10 (16.9)	12 (17.6)	11 (16.9)
Once a week	10 (14.9)	9 (15.3)	8 (11.8)	8 (12.3)	9 (13.4)	8 (13.6)	12 (17.6)	12 (18.5)
More seldom/never	32 (47.8)	29 (49.2)	34 (50.0)	32 (49.2)	19 (28.4)	16 (27.1)	21 (30.9)	19 (29.9)
Toothpick use								
≥ 3 times/day	1 (1.5)	1 (1.7)	1 (1.5)	1 (1.5)	3 (4.5)	3 (5.1)	0	0
Twice a day	1 (1.5)	1 (1.7)	2 (2.9)	2 (3.1)	4 (6.0)	4 (6.8)	2 (2.9)	2 (3.1)
Once a day	2 (3.0)	2 (3.4)	7 (10.3)	6 (9.2)	7 (10.4)	7 (11.9)	11 (16.2)	10 (15.4)
Several times/week	6 (9.0)	6 (10.2)	7 (10.3)	7 (10.8)	5 (7.5)	5 (8.5)	6 (8.8)	6 (9.2)
Once a week	4 (6.0)	3 (5.1)	3 (4.4)	2 (3.1)	5 (7.5)	4 (6.8)	6 (8.8)	5 (7.7)
More seldom/never	53 (79.1)	46 (78.0)	48 (70.6)	47 (72.3)	43 (64.2)	36 (61.0)	43 (63.2)	42 (64.6)
Additional fluoride								
≥ 3 times/day	3 (4.5)	3 (5.1)	4 (5.9)	3 (4.6)	5 (7.5)	5 (8.5)	3 (4.4)	2 (3.1)
2 times/day	13 (19.4)	11 (18.6)	8 (11.8)	7 (10.8)	19 (28.4)	17 (28.8)	14 (20.6)	13 (20.0)
Once a day	12 (17.9)	11 (18.6)	14 (20.6)	14 (21.5)	13 (19.4)	12 (20.3)	17 (25.0)	17 (26.2)
Several times/week	10 (14.9)	8 (13.6)	13 (19.1)	13 (20.0)	16 (23.9)	14 (23.7)	18 (26.5)	18 (27.7)
Once a week	10 (14.9)	9 (15.3)	10 (14.7)	10 (15.4)	4 (6.0)	3 (5.1)	6 (8.8)	6 (9.2)
More seldom/never	19 (28.4)	17 (28.8)	19 (27.9)	18 (27.7)	10 (14.9)	8 (13.6)	10 (14.7)	9 (13.8)

Frequency n (%)

## Results

### Description of participants at baseline

In total, 135 individuals were included in the study, and were allocated to either intervention (*n* = 67) or control (*n* = 68). Sociodemographic and clinical characteristics of the participants are presented in Table 2. The mean age was 20 years, and the participants had a mean number of caries surfaces of 6.3 and 4.9 in the intervention and control group, respectively. The vast majority of participants experienced their oral health to be poor or fair, with 86.6% and 82.4% in the intervention and control group, respectively. About 40% in both groups rated their oral health as poor, less than 20% rated their oral health as good, and none rated it as very good. Half of the subjects were female, one third was smokers, and different ethnicities and socioeconomic positions were represented in the study group. The intervention group reported statistically significantly more Swedish-born mothers than the control group (65.7% vs. 42.6%, *p* < 0.01 (ns. after Bonferroni correction)), while the groups did not differ with regard to the other sociodemographic and clinical measures. The study groups did not differ with regard to oral health behaviour variables at baseline (Table 3).

### Changes after intervention

The number of participants who received intended treatment and were analysed, as well as participant losses after randomization, are presented in Fig. 1. In the intervention group, 64 individuals received the allocated treatment and 59 of them participated in the follow-up, while 68 individuals received the control condition, and 65 of them participated in the follow-up. Per protocol analyses revealed that the intervention group improved their oral health behaviour on all four measures (Table 3): tooth-brushing (Z = − 3.43, *p* = 0.001, effect size 0.32); flossing (Z = − 3.48, *p* = 0.0005, effect size 0.32); toothpicks (Z = − 3.04 *p* = 0.002, effect size 0.28); additional use of fluoride (Z = − 3.27 *p* = 0.001, effect size 0.30). The control group improved their oral health behaviour regarding two variables: use of flossing (Z = − 2.72, *p* = 0.006 (ns. after Bonferroni correction), effect size 0.24) and additional use of fluoride (Z = − 2.53, *p* = 0.011 (ns. after Bonferroni correction), effect size 0.22), while no differences were found regarding tooth-brushing (Z = − 0.99, *p* = 0.320) and toothpicks (Z = − 0.73, *p* = 0.466).

Intention-to-treat analyses showed parallel results in that the intervention group improved their oral health behaviour on all four measures (Table 3): tooth-brushing (Z = − 3.43, *p* = 0.001, effect size 0.30); flossing (Z = − 3.48, *p* = 0.0005, effect size 0.30); toothpicks (Z = − 3.04 *p* = 0.002, effect size 0.26); additional use of fluoride (Z = − 3.27 p = 0.001, effect size 0.28). The control group improved their oral health behaviour over time concerning two variables: use of flossing (Z = − 2.72, *p* = 0.006 (ns. after Bonferroni correction), effect size 0.23), and additional use of fluoride (Z = − 2.53, *p* = 0.011 (ns. after Bonferroni correction), effect size 0.22), while no differences were found concerning tooth-brushing (Z = − 0.99, *p* = 0.320) and toothpicks (Z = − 0.73, *p* = 0.466).

No adverse events were reported by the participants. The period of recruitment of participants to final examination at follow-up lasted between February 2013 and May 2016.

## Discussion

This randomised controlled trial evaluated the effect of a brief psychological intervention (ACT) for behaviour change, delivered by a psychologist in general dental care to young adults (18–25 years of age) with dental caries. Significant positive changes with regard to oral health behaviours were found, most prominent in the intervention group compared with the control group that received standardised information. However, the hypothesis stated was only accepted in part regarding the measures of oral health behaviours (i.e., the ACT intervention improved oral health behaviours more than information alone).

There are mixed results in the literature on behavioural interventions to improve oral health behaviour in individuals with poor oral health [7, 24]. Positive effects on tooth-brushing and interdental cleaning have been reported in studies with an RCT design including middle-aged to older individuals with periodontitis [25–27]. In the present study on young adults (18–25 years of age) with dental caries, the intervention group improved their oral health behaviour on all investigated variables (tooth-brushing, flossing, use of toothpicks and additional use of fluoride). This is a promising result. The participants in this study were all affected by severe dental caries disease, and behavioural change was necessary to halt the disease progression and to promote better oral health.

The control group also showed some improvement in oral health behaviour, although on fewer measures. There are potential general effects of being a participant in a clinical study, such as receiving extra attention from dental personnel, which may contribute to positive changes also in the control group. It is not reasonable to argue that the control condition in itself led to these changes in the control group, since the control condition consisted of the ordinary treatment-as-usual information delivered to all patients.

Previous studies on psychological interventions for behavioural change in the area of dentistry have mainly focused on interventions inspired of or based on the Motivational Interviewing technique, applied to patients with periodontitis at specialised clinics [26, 28–30]. Interventions based on other theoretical models have also been presented [27, 31, 32]. This study adds important knowledge to the field by testing ACT, a theory-based psychological intervention used with promising results for various health issues in health care [13, 14] but, to our knowledge, not previously used in dentistry. With regard to ACT, one may specifically emphasize certain modules in the first session, such as the brief interview and focused questions leading to the case conceptualisation, providing information and stance for individualised interventions to increase psychological flexibility and contact with life values. However, we believe that the all-embracing model of ACT as a specific type of CBT-intervention is the most important factor for the behaviour changes accomplished.

The present study also used a multi-professional setting, where dental personnel identified eligible participants, and where the intervention was delivered by a licensed psychologist working at the same general dental clinic. Over the last decades it has become more common to include psychologists in primary care settings [33]. The same development has not taken place within dentistry, with the exception of treatment of patients with dental phobia, where psychologists are members of treatment teams in many specialised clinics [34–36].

In this paper we have discussed the effect of ACT on oral health behaviour. Other behavioural outcomes of relevance for oral health are for example tobacco use and dietary habits. Behaviour change interventions or counselling has proven effective for tobacco use cessation in adults in both general medicine and dentistry [37]. However, according to a Cochrane review [38], there is limited evidence about effective interventions (behavioural and/or medical) for smoking cessation in young people. When it comes to dietary habits, there is evidence from the field of general medicine that behaviour change or counselling could effectively change such habits [37]. Yet, in dentistry, such interventions have only had limited effect. In fact, as mentioned previously, a recent systematic review on interventions for dietary change in adult patients with dental caries found no such studies [9]. Thus, there are several knowledge gaps to address in the future.

This study has some strengths and limitations. The study used an appropriate RCT design while adhering to the standard protocol for such a design, according to the CONSORT methodology. We included a large number of participants at baseline and had a dropout rate of only 15.5% at follow-up. Moreover, the analyses included both per protocol and intention-to-treat evaluations. The study group of young adults, between the ages of 18 and 25 years, is in a period of their lives when mobility is common. Individuals move away from home, find employment or may enrol in higher education; thus, an even greater loss to follow-up was expected. The generalisability of the study is high, as the study was conducted in two general Public Dental Service clinics. In Sweden, the large majority of individuals in this age-group regularly visit Public Dental Service clinics. The participants were recruited while on their ordinary visit at the clinics, where registrations and interventions were performed. A desirable double-blind procedure was obviously not possible, due to the design and intervention tested. However, we were able to blind the research group and the statistician to which group the participants belonged. The outcome measures are self-reported only and it is therefore important to include objective clinical health measurements, such as gingivitis and caries. Even if the results are promising with regard to oral health behaviour, we need to conclude on the long-term effects of the psychological intervention, i.e. the sustainability of the results.

To the best of our knowledge, while searching the scientific literature, we have not found other RCTs testing a behavioural intervention on young adults (18–25 years of age) with high caries activity, nor do we know of a similar field study setting where a licensed psychologist has been employed within general dentistry clinics to treat young adults affected by caries disease. It may be argued that the dental professions need other professionals, such as psychologists, when treating or counselling young adults in order to alter their health behaviour related to different oral diseases. Moreover, this is particularly important considering the often high prevalence of oral diseases, the close relationship between oral diseases and health behaviour, and the fact that these diseases, in terms of etiologic fraction, are highly preventable.

## Conclusions

By testing a psychological intervention (Acceptance and Commitment Therapy) on young adults (18–25 years of age) with high caries prevalence, we found an immediate positive effect with improved oral health behaviours, including more tooth-brushing, flossing, and the use of toothpicks and additional use of fluoride.

## Abbreviations

ACT: Acceptance and Commitment Therapy
CBT: Cognitive Behaviour Therapy
ITT: Intention-to-treat
ns.: Non-significant
PP: Per protocol
